# D-galacto-D-mannan-mediated Dectin-2 activation orchestrates potent cellular and humoral immunity as a viral vaccine adjuvant

**DOI:** 10.3389/fimmu.2024.1330677

**Published:** 2024-02-16

**Authors:** Hyeong Won Kim, Mi-Kyeong Ko, So Hui Park, Seokwon Shin, Gang Sik Kim, Dong Yun Kwak, Jong-Hyeon Park, Su-Mi Kim, Jong-Soo Lee, Min Ja Lee

**Affiliations:** ^1^ Center for Foot-and-Mouth Disease Vaccine Research, Animal and Plant Quarantine Agency, Gimcheon, Gyeongsangbuk-do, Republic of Korea; ^2^ College of Veterinary Medicine, Chungnam National University, Daejeon, Republic of Korea

**Keywords:** foot-and-mouth disease, D-galacto-D-mannan, Dectin-2 agonist, vaccine adjuvant, cellular and humoral, immune response

## Abstract

**Introduction:**

Conventional foot-and-mouth disease (FMD) vaccines have been developed to enhance their effectiveness; however, several drawbacks remain, such as slow induction of antibody titers, short-lived immune response, and local side effects at the vaccination site. Therefore, we created a novel FMD vaccine that simultaneously induces cellular and humoral immune responses using the Dectin-2 agonist, D-galacto-D-mannan, as an adjuvant.

**Methods:**

We evaluated the innate and adaptive (cellular and humoral) immune responses elicited by the novel FMD vaccine and elucidated the signaling pathway involved both *in vitro* and *in vivo* using mice and pigs, as well as immune cells derived from these animals.

**Results:**

D-galacto-D-mannan elicited early, mid-, and long-term immunity *via* simultaneous induction of cellular and humoral immune responses by promoting the expression of immunoregulatory molecules. D-galacto-D-mannan also enhanced the immune response and coordinated vaccine-mediated immune response by suppressing genes associated with excessive inflammatory responses, such as nuclear factor kappa B, *via* Sirtuin 1 expression.

**Conclusion:**

Our findings elucidated the immunological mechanisms induced by D-galacto-D-mannan, suggesting a background for the robust cellular and humoral immune responses induced by FMD vaccines containing D-galacto-D-mannan. Our study will help to facilitate the improvement of conventional FMD vaccines and the design of next-generation FMD vaccines.

## 1 Introduction

Foot-and-mouth disease (FMD) is a highly contagious viral disease of livestock that causes significant economic losses. The FMD virus (FMDV) belongs to the *Picornaviridae* and *Aphthovirus* families and is classified into seven serotypes (1, 2). The inability of cross-protection among the seven FMDV serotypes complicates its prevention and control (3, 4). FMD is typically identified by symptoms, such as high fever, blisters in the mouth, and excessive secretion of sticky or foamy saliva (5). Additionally, adult animals can experience weight loss that they do not recover for several months, swelling in male testicles, and significant reduction in cattle milk production. Although several infected animals remain asymptomatic carriers, they can carry the virus and transmit it to other animals (6, 7).

Many countries have recommended vaccination to prevent FMD from acute spreading; however, available vaccines have several limitations, such as low antibody titers and local reactions at the injection site. Therefore, we investigated effective adjuvants to enhance the cellular and humoral immune responses of the vaccine and address safety concerns. Korea belongs to FMDV serotype pool 1 and is mainly exposed to FMDV serotypes O, A, and Asia 1 (8). Since 2000, FMD outbreaks in Korea have been primarily attributed to serotypes O and A. Indeed, recent outbreaks of FMD in Korea from 2017 to 2023 were caused by type O (ME-SA topotype) and type A (A/ASIA/Sea-97 topotype). Therefore, in this study, a test vaccine was prepared using FMD antigens O PA2 (ME-SA topotype) and A YC (A/ASIA/Sea-97 topotype).

Adjuvants enhance and prolong the immune response when used in combination with specific vaccine antigens (9); therefore, to develop a novel FMD vaccine, research on various adjuvants must be conducted.

Most FMD vaccines involve using inactivated viral antigens. Mineral oil-based adjuvants and aluminum hydroxide [Al(OH)_3_] with or without saponin have been used as traditional adjuvants for FMD vaccines to improve the stability and delivery of inactivated viral antigens (10–13). Several problems have been reported with FMD vaccines containing crude saponins, including hemolysis at the vaccination site and eliciting short-lived antibody responses. Therefore, Quil-A, which is safer than saponin and can induce a strong immune response, was used as an FMD vaccine adjuvant (14).

Despite improved FMD vaccines, repeated vaccination is recommended owing to low and short-lived antibody titers. Repeated vaccinations may cause local side effects at the injection site due to the mineral oil-based adjuvant contained in the FMD vaccine (11, 13, 15–17).

Thus, adjuvants, specifically immunostimulant combinations currently used in FMD vaccines, require improvement to enhance efficacy and safety. In a previous study, we confirmed that treatment of porcine peripheral blood mononuclear cells (PBMCs) with a dendritic cell (DC)-associated C-type lectin-2 (Dectin-2) agonist induced PBMC proliferation (18). Therefore, we hypothesized that Dectin-2 activation elicited a robust immune response in pigs.

Based on previous studies, we used D-galacto-D-mannan, a Dectin-2 agonist, as an adjuvant for the novel FMD vaccine in this study. Dectin-2 is a pattern recognition receptor (PRR) containing a C-type-like lectin domain with a mannose-binding glutamic acid-proline-asparagine motif that binds to high mannose-containing structures (19). Immunomodulatory responses through Dectin-2 have been reported in FMD-susceptible animals, including sheep and cattle (20, 21).

Dectin-2 is expressed in cells, including Langerhans cells, macrophages (MΦs), neutrophils, and several DC subsets (22, 23). Dectin-2 signaling is induced through the spleen tyrosine kinase (SYK), protein kinase C delta (PKCδ), and caspase-associated recruitment domain 9 (CARD9)-B-cell lymphoma 10 (BCL10)-mucosa-associated lymphoid tissue lymphoma translocation gene 1 (MALT1) downstream pathways, inducing several cytokines, such as interleukin (IL)-2, IL-10, IL-1β, IL-6, IL-12, and IL-23 (24, 25).

Dectin-2 signals also include phospholipase Cγ2 and mitogen-activated protein kinases, which selectively stimulate the c-Rel, nuclear factor kappa B (NF-κB) subunit through MALT1 to promote the secretion of T helper 17 (Th17) polar cytokines (IL-1β and IL-23) (26). Dectin-2 signaling also promotes the Th2 immune response by inducing cysteinyl leukotriene production (27). Considering these characteristics, Dectin-2 was selected among many PRRs and assessed as a target; D-galacto-D-mannan, a polysaccharide composed of a mannose backbone and galactose side group, was used as a Dectin-2 agonist. D-galacto-D-mannan, a hemicellulose derived from plant cell walls, has antioxidant activity against hydroxyl radical production (28). In this study, we assessed the innate and adaptive (cellular and humoral) immune responses induced by a novel FMD vaccine in mice and pigs, as well as immune cells derived from these animals (*in vitro* and *in vivo*), and elucidated the signaling pathway involved.

## 2 Materials and methods

### 2.1 Antigen purification

Purified antigens were prepared from BHK-21 (C-13) [baby hamster kidney; American Type Culture Collection (ATCC), VA, USA] cells infected with FMDV O PA2 (GenBank Accession No. AY593829.1) and FMDV A YC (GenBank Accession No. KY766148.1), as described previously (7, 29). For viral infection, O PA2 and A YC were inoculated into BHK-21 cells in serum-free culture medium—Dulbecco’s modified Eagle’s medium (DMEM; HyClone, UT, USA) and incubated at 37°C and 5% CO_2_ for 1 h. Extracellular viruses were removed; 16 h after infection, the virus was inactivated by treatment with 0.003 N binary ethylenimine (BEI) twice. The inactivated virus was precipitated with polyethylene glycol 6000 (Sigma-Aldrich, MO, USA) (30). Antigen (146S) was purified using a 15%–45% sucrose density gradient and ultracentrifuged. Approximate quantities of FMDV antigen were confirmed *via* optical density measurements using a lateral flow device (BioSign FMDV Ag; Princeton BioMeditech, NJ, USA). Prior to experimental use, the BEI-treated supernatant was confirmed to be free of live viruses with inactivation tests using ZZ-R 127 [fetal goat tongue epithelium; the Collection of Cell Lines in Veterinary Medicine (CCLV), Friedrich-Loeffler-Institut, Greifswald-Insel Riems, Germany)] and BHK-21 cells (7, 29).

### 2.2 Cell viability assay

BHK-21, LF-BK (fetal porcine kidney; Plum Island Animal Disease Center, NY, USA), and ZZ-R cells (2 × 10^4^ cells/well) were treated and cultured for 48 h. Purified peritoneal exudate cells (PECs) and PBMCs (1 × 10^5^ cells/well) were treated and stabilized for 1 h. D-galacto-D-mannan (0, 0.625, 1.25, 2.5, or 5 μg/mL; Sigma-Aldrich) was then added and cultured for 4 h. Cell viability was measured with an MTS (inner salt)-based colorimetric assay (Promega, WI, USA) following the manufacturer’s instruction. Data were obtained using a Hidex 300SL spectrophotometer (Hidex, Turku, Finland) at 490 nm.

### 2.3 Animals

Mice (experimental animals) and pigs (target animals) were managed as previously described (7, 29). Mice (C57BL/6, females, 6–7 weeks old) were purchased from KOSA BIO Inc. (Gyeonggi, Korea). Farm pigs (Landrace, 8–9 weeks old) that were negative for FMDV type O and A antibodies [using SP enzyme-linked immunosorbent assay (ELISA) in serum] were used. During the study, all animals were housed in a specific pathogen-free animal biosafety level 3 facility at the Animal and Plant Quarantine Agency (APQA) and were used for the experiment after at least 1 week of adaptation. The study and all experimental protocols were approved by the APQA Ethics Committee (Certification No.: IACUC-2022-670 and 2023-753).

### 2.4 PEC and PBMC isolation

For PEC isolation, naive mice (total *n* = 10) were anesthetized with CO_2_ and euthanized as previously described (7, 29). The abdominal cavity was flushed with 3 mL of Ca^2+^/Mg^2+^-free Dulbecco’s phosphate-buffered saline (DPBS; Gibco, MA, USA). The collected peritoneal lavage fluid was centrifuged, and the pelleted PECs were resuspended and counted using an automated cell counter (Bio-Rad TC20; Bio-Rad Laboratories, CA, USA). In particular, cryopreserved cells were not used in any experiments.

For PBMC isolation, whole blood of pigs (*n* = 5–6/group) was used as previously described (7, 29). Whole blood (10 mL/donor) was individually collected, and PBMCs were isolated using Lymphoprep™ (Stem Cell Technologies, BC, Canada). Red blood cells were lysed with ammonium–chloride–potassium (ACK) lysing buffer (Gibco). PBMCs were suspended in Ca^2+^/Mg^2+^-free Dulbecco’s PBS (Gibco) and analyzed using the automated cell counter (Bio-Rad). No cells used in the experiments were cryopreserved. Isolated PECs and PBMCs were incubated in RPMI1640 (Gibco) medium (7, 29).

### 2.5 Interferon γ enzyme-linked immnuosorbent spot assay

D-galacto-D-mannan-mediated interferon γ (IFNγ) secretion with or without antigen (O PA2 or A YC) was assayed using commercial enzyme-linked immune absorbent spot (ELISpot) assay kits (R&D Systems, MN, USA) as per the manufacturer’s recommendations. Murine PECs or porcine PBMCs (5 × 10^5^ cells/well) were cultured in 96-well polyvinylidene fluoride-backed microplates containing a monoclonal capture antibody specific for murine or porcine IFNγ and stimulated with 2 μg/mL (final concentration) of antigen mixed with 0.625, 1.25, 2.5, and 5 μg/mL of D-galacto-D-mannan sequentially, at 37°C and 5% CO_2_ for 18 h. Antigen and PBS were used as positive (PC) and negative controls (NC), respectively. Data were obtained using the ImmunoSpot ELISpot reader (AID iSpot Reader System; Autoimmune Diagnostika GmbH, Strassberg, Germany). The results were presented as spot-forming cells (SFC) per number of cells added to the well (7, 29).

### 2.6 Assessment of early host defense in mice administered with D-galacto-D-mannan alone

Before evaluating the potential of D-galacto-D-mannan as an FMD vaccine adjuvant, the potential of D-galacto-D-mannan alone to elicit host protection against FMDV infection was evaluated.

The experimental (Exp) group mice received 100 μg of D-galacto-D-mannan/dose/mouse for a total volume of 100 μL, while the NC group received an equal volume of PBS. Mice (*n* = 5/group) were administered intramuscular (IM) injection [0 days post-injection (dpi)] and challenged with FMDV (100 LD_50_ of O/VET/2013 or 100 LD_50_ A/Malay/97) *via* intraperitoneal (IP) injection, 3 or 7 dpi. Survival rates and body weight were monitored for up to 7 days post-challenge (dpc).

### 2.7 Evaluation of safety in mice vaccinated with the FMD vaccine containing D-galacto-D-mannan

The safety of the FMD vaccine containing D-galacto-D-mannan was evaluated in mice as previously described (29). The vaccine compositions for the usual vaccination in mice were as follows: purified antigens obtained *via* antigen purification from FMDV type O (O PA2) and type A (A YC) (0.375 μg + 0.375 μg/dose), ISA 206 (Seppic, Paris, France; 50% w/w), 10% aluminum hydroxide [Al(OH)_3_], and 15 μg/mouse Quil-A (InvivoGen, CA, USA), with the addition of 100 μg of D-galacto-D-mannan/dose/mouse in a total volume of 100 μL. Mice were administered with a vaccine equivalent to fivefold (500 μL) the volume of the usual vaccination dose (100 μL). All mice (*n* = 5/group) were vaccinated with IP injection into the peritoneum (0 dpi). To evaluate the safety of the vaccines, survival rates and body weight changes were evaluated up to 7 dpi.

### 2.8 Evaluation of early host defense in mice immunized with FMD vaccine containing D-galacto-D-mannan as an adjuvant

The protective effect of FMD vaccine containing D-galacto-D-mannan was then verified in the early stages of viral infection in mice (*n* = 5/group). The vaccine formula for the PC group was as follows: purified antigens type O (O PA2) and type A (A YC) (0.375 μg + 0.375 μg/dose), 10% Al(OH)_3_, ISA 206 (Seppic; 50% w/w), and 15 μg/dose/mouse Quil-A (InvivoGen), in a total volume of 100 μL. The Exp group received vaccines with the same formula as the PC group, with the addition of 100 μg of D-galacto-D-mannan/dose/mouse as an adjuvant, *via* the same route. The NC group received an equal volume of PBS. Mice were vaccinated *via* IM injection [0 days post-vaccination (dpv)] and challenged with FMDV (100 LD_50_ of O/VET/2013 or 100 LD_50_ A/Malay/97) *via* IP injection 7 dpv. Survival rates and body weight were monitored for up to 7 dpc.

### 2.9 Evaluation of early, mid-, and long-term immune response of FMD vaccines including D-galacto-D-mannan as an adjuvant in mice

Early, mid-, and long-term immune responses were monitored in mice to evaluate the efficacy of the FMD vaccine containing D-galacto-D-mannan. The test vaccine formula was the same as described in section 2.7. Mice (*n* = 5/group) were immunized with the test vaccine and blood samples were collected 0, 7 (early), 28 (mid-term), 56, and 84 (long-term) dpv for serological analysis. Serum samples were stored at −80°C until analysis.

### 2.10 Evaluation of early, mid-, and long-term immune responses to FMD vaccine containing D-galacto-D-mannan in pigs

To assess the induction of early, mid-, and long-term immune responses of the FMD vaccine with D-galacto-D-mannan in target animals, a study was conducted as previously described (7, 29). Animals were randomly divided into three groups; NC, PC, and Exp group (*n* = 5–6/group). The vaccine formula for the PC group was as follows: purified antigens type O (O PA2) and type A (A YC) (15 + 15 μg/dose/mL), 10% Al(OH)_3_, ISA 206 (Seppic; 50% w/w), and 150 μg/dose/pig Quil-A (InvivoGen), in a total volume of 1 mL. The Exp group received test vaccines with the same formula as the PC group, with the addition of 1 mg of D-galacto-D-mannan/dose/pig as an adjuvant, *via* the same route. NC group pigs were administered an equal volume of PBS. After primary vaccination *via* IM injection, a booster shot was administered *via* the same route 28 dpv. Sera were collected from the vaccinated pigs 0, 7, 14 (early), 28, 42 (mid-term), 56, and 84 (long-term) dpv for serological assays. The local reaction was confirmed at the vaccination site (neck) by sacrificing pigs at 84 dpv. Under the observation of a veterinarian, both sides of the neck were cut into circular shapes with a diameter of 20 cm and a length of 40 cm and cut into 1.5-cm-thick pieces; the effect of suppressing abnormal meat formation was confirmed through visual inspection.

### 2.11 Serological assays

To detect SP antibodies in serum samples, VDPro^®^ FMDV type O kit (Median Diagnostics, Gangwon-do, Korea) and PrioCheck™ FMDV type A kit (Prionics AG, Schlieren, Switzerland) were used as per the manufacturer’s instructions. Since the FMDV antigen subtypes coated on the VDPro^®^ and PrioCheck™ kits were different, antibody titer was measured using both kits to prevent underestimation of the characteristics of the antigen coating the kit. Data were obtained using a spectrophotometer at 450 nm (7, 29), and the absorbance was converted to the percent inhibition (PI) value. When the PI value was ≥40% for the VDPro^®^ FMDV kit or ≥50% for the PrioCheck™ FMDV kit, the animals were considered antibody-positive.

The VN test was conducted following the guidelines of the World Organization for Animal Health (31). Sera samples were heat-inactivated, diluted, and incubated with a 100 50% tissue culture infective dose (TCID_50_) in 50 μL of FMDV (O PA2 or A YC) media at 37°C for 1 h. Subsequently, 50 μL of LF-BK cells (10^6^ cells/mL) was added to each well and incubated at 37°C and 5% CO_2_ for 3 days, and the wells were checked for cytopathic effects. VN titers were evaluated as the Log_10_ of the reciprocal antibody dilution required for neutralization of 100 TCID_50_ of viruses in 50% of the wells (32).

To detect antigen-specific antibodies [immunoglobulin (Ig) subtype], ELISAs were performed for porcine IgM, IgA, and IgG (Bethyl Laboratories Inc., TX, USA) on the serum samples, as per the manufacturer’s instructions. Data were obtained using a spectrophotometer at 450 nm (7, 29).

### 2.12 RNA isolation, cDNA synthesis, and quantitative real-time polymerase chain reaction

To investigate the immune response mechanism elicited by the FMD vaccine with D-galacto-D-mannan, an experiment was performed according to the previously described protocol (7, 29). Total RNA was extracted using TRIzol reagent (Invitrogen, CA, USA) and Rneasy Mini Kit (QIAGEN, CA, USA) as per the manufacturer’s instructions. Complementary DNA (cDNA) was prepared *via* reverse transcription using the GoScript Reverse Transcription System (Promega) as per the manufacturer’s recommendations. Synthesized cDNAs were amplified using quantitative real-time polymerase chain reaction (qRT-PCR) on a Bio-Rad iCycler using iQ SYBR Green Supermix (Bio-Rad). Gene expression levels were normalized to HPRT (reference gene) levels and presented as relative ratios compared with the control (29). The primers used in this study are listed in **Supplementary Table 1**.

### 2.13 Evaluation of the efficacy of induction of systemic and mucosal immunity and the sustainability of long-lasting immune responses through the combination of intramuscular vaccination of FMD vaccine and oral administration of D-galacto-D-mannan

To verify the effect of simultaneously inducing systemic immunity and mucosal immunity through a combined program of intramuscular vaccination of FMD vaccine and oral administration of D-galacto-D-mannan, an experiment was performed in mice (*n* = 5/group). The vaccine formula for the PC group was as follows: purified antigens type O (O PA2) (0.375 μg/dose), 10% Al(OH)_3_, ISA 206 (Seppic; 50% w/w), and 15 μg/dose/mouse Quil-A (InvivoGen), in a total volume of 100 μL. The Exp group received vaccines with the same formula as the PC group *via* the same route, and administered orally 100 μg of D-galacto-D-mannan/dose/mouse in 100 μL of PBS. The NC group received an equal volume of PBS. Mice were vaccinated *via* IM injection (0 dpv) and then received D-galacto-D-mannan or PBS orally daily until 28 dpv. Serological assays such as antibody titers by SP O ELISA and VN titers by VN test were performed as described in section 2.11.

### 2.14 Statistical analysis

Quantitative data are expressed as the mean ± standard error unless otherwise stated. Between-group statistical differences were assessed using a two-way or one-way analysis of variance, followed by Tukey’s or Dunnett’s *post-hoc* test. Statistical significance was denoted as ^*^ *p* < 0.05, ^**^ *p* < 0.01, ^***^ *p* < 0.001, and ^****^ *p* < 0.0001. Parametric tests were used to compare the different groups. Survival curves were constructed using the Kaplan–Meier method, and differences were analyzed using the log-rank sum test. Prism 10.0.2 (GraphPad, San Diego, CA, USA) was used for all statistical analyses.

## 3 Results

### 3.1 D-galacto-D-mannan induces potent innate and adaptive (cellular) immune response *via* IFNγ expression in PECs and PBMCs *in vitro* and exhibits an adjuvant effect when combined with an inactivated FMDV antigen

**Figure 1:**
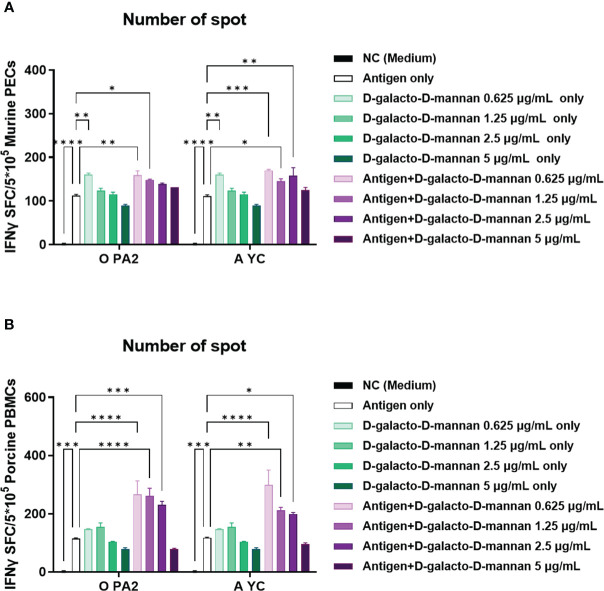
D-galacto-D-mannan with FMDV type O (O PA2) or A (A YC) antigen induces potent innate and adaptive (cellular) immune responses in murine PECs and porcine PBMCs. IFNγ secretion mediated by O PA2 or A YC antigen, with or without D-galacto-D-mannan, was evaluated using ELISpot assay in murine PECs and porcine PBMCs. Data are presented as spot-forming cells per number of cells in the well and mean ± SEM of triplicate measurements (*n* = 3/group). **(A, B)** IFNγ-secreting cell spots in murine PECs **(A)** and porcine PBMCs **(B)**. Statistical analyses were performed using one-way analysis of variance followed by Tukey’s *post-hoc* test. *p < 0.05; **p < 0.01; ***p < 0.001; ****p < 0.0001. FMDV, foot-and-mouth disease virus; PECs, peritoneal exudate cells; PBMCs, peripheral blood mononuclear cells; IFNγ, interferon γ.

Before conducting the study, we confirmed the cytotoxicity of D-galacto-D-mannan through *in vitro* experiments (**Supplementary Figures 1A–E**) and observed no cytotoxicity at the concentration of ≤5 μg/mL of D-galacto-D-mannan in tested cells. In an *in vitro* ELISpot assay performed using PECs isolated from mice, the Exp group containing D-galacto-D-mannan showed a more significant IFNγ secretion than the PC group administered with antigens (type O or A) or D-galacto-D-mannan alone (**Figure 1A**, **Supplementary Figure 2A**). In the *in vitro* ELISpot assay performed using PBMCs isolated from porcine whole blood, the Exp group containing D-galacto-D-mannan showed a significantly higher IFNγ secretion than the PC group administered with antigen or D-galacto-D-mannan alone (**Figure 1B**, **Supplementary Figure 2B**). These results demonstrated that D-galacto-D-mannan, used as an FMD vaccine adjuvant, enhances innate and cellular immune responses in mice and pigs.

### 3.2 The FMD vaccine containing D-galacto-D-mannan as an adjuvant protects the host during the early stages of viral infection in mice

Before evaluating the host protection in mice of the FMD vaccine containing D-galacto-D-mannan as an adjuvant, we investigated D-galacto-D-mannan-mediated host defense. However, D-galacto-D-mannan alone did not elicit host protection in mice (**Supplementary Figures 3A–I**). To investigate the safety of vaccine containing D-galacto-D-mannan, we administered a 500 μL (100 μL × 5-fold) intraperitoneal (I.P.) injection of an FMD vaccine containing D-galacto-D-mannan into the mouse peritoneal cavity and monitored it for up to 7 dpv. We found that all mice achieved a 100% survival rate, indicating that the FMD vaccine containing D-galacto-D-mannan as an adjuvant was safe (**Supplementary Figures 4A, B**).

**Figure 2:**
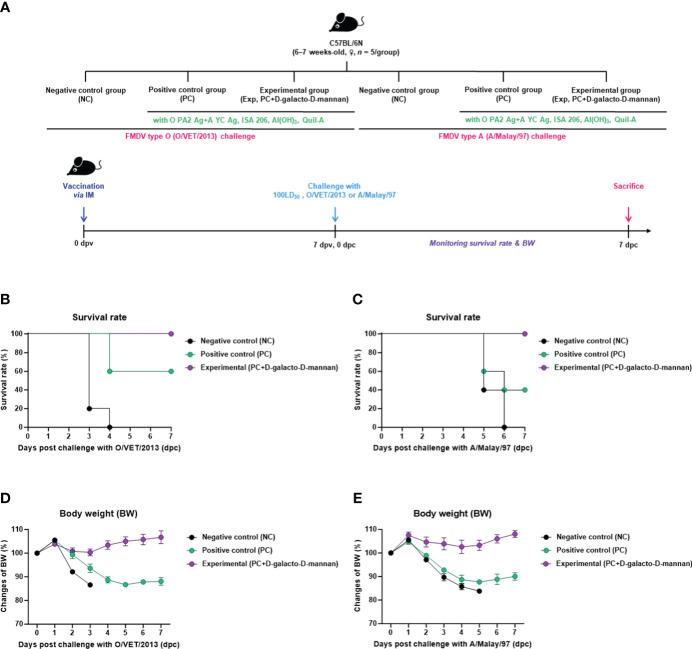
FMD vaccine containing D-galacto-D-mannan enhances vaccine efficacy and protective effects during the early stages of viral infection in mice. C57BL/6 mice (*n* = 5/group) were administered FMD vaccine containing inactivated FMDV antigens isolated from FMDV type O (O PA2) and A (A YC) (0.375 + 0.375 μg/dose/100 μL; 1/40 of the dose for pigs), 100 μg of D-galacto-D-mannan/dose/mouse, ISA 206 (oil-based emulsion, 50%, w/w), 10% aluminum hydroxide, and 15 μg of Quil-A. The PC group received vaccines of the same volume and formula as the Exp group but without D-galacto-D-mannan as an adjuvant. The NC group was injected with an equal volume of phosphate-buffered saline. Test vaccines were injected intramuscularly into mice later challenged with FMDV O (100 LD_50_ O/VET/2013) or FMDV A (100 LD_50_ A/Malay/97) 7 dpv *via* an intraperitoneal injection. Survival rates and body weights were monitored for 7 days post-challenge. **(A–E)** Experimental strategy **(A)**; survival rates post-challenge with O/VET/2013 **(B)** and A/Malay/97 **(C)**; body weight changes post-challenge with O/VET/2013 **(D)** and A/Malay/97 **(E)**. Data are represented as mean ± SEM of triplicate measurements (*n* = 5/group). FMD, foot-and-mouth disease; FMDV, foot-and-mouth disease virus; PC, positive control; NC, negative control; dpv, days post-vaccination.

To evaluate the initial host defense of the FMD vaccine containing D-galacto-D-mannan as an adjuvant against FMDV infection, experiments were conducted following the strategy presented in **Figure 2A**. A bivalent vaccine (with O PA2 + A YC antigen) containing D-galacto-D-mannan showed 100% survival rates against O/VET/2013 and A/Malay/97 (**Figures 2B, C**). Body weights of mice did not significantly change in the Exp group receiving the D-galacto-D-mannan-containing vaccine (**Figures 2D, E**). In the PC group not receiving D-galacto-D-mannan, the survival rate of FMDV type O and A infections were 60% and 40%, respectively, and the body weight decreased by more than 10% at 4 dpc. In the NC group, the mortality rate was 100% (survival rate 0%) 4 and 6 dpc for FMDV type O and A challenges, respectively. These results demonstrate that D-galacto-D-mannan plays a critical role in initial host defense in mice.

### 3.3 FMD vaccine containing D-galacto-D-mannan as an adjuvant elicits potent early, mid-, and long-term immunity in mice and pigs

**Figure 3:**
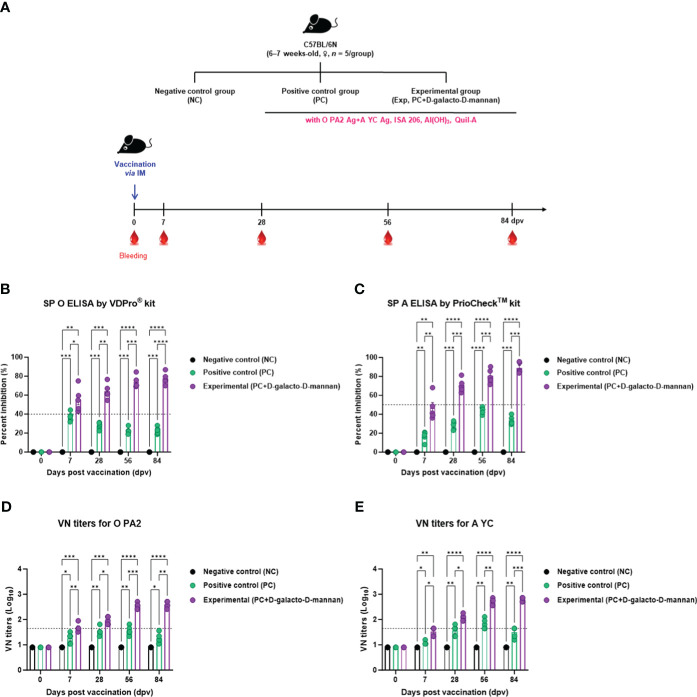
FMD vaccine containing D-galacto-D-mannan elicits early, mid-, and long-term immune response in mice. C57BL/6 mice (*n* = 5/group) were administered FMD vaccine containing inactivated FMDV antigens isolated from FMDV type O (O PA2) and A (A YC) (0.375 + 0.375 μg/dose/100 μL; 1/40 of the dose for pigs, 100 μg of D-galacto-D-mannan/dose/mouse, ISA 206 (oil-based emulsion, 50%, w/w), 10% aluminum hydroxide, and 15 μg of Quil-A. The PC group received vaccines of the same volume and formula as the Exp group, but without D-galacto-D-mannan as an adjuvant. The NC group was injected with an equal volume of phosphate-buffered saline. Mice were vaccinated with test vaccine intramuscularly and blood was collected 0, 7, 28, 56, and 84 dpv for serological analysis using SP O and A ELISAs and VN titers for O/PKA/44/2008 (O PA2) and A/SKR/YC/2017 (A YC). **(A–E)** Experimental strategy **(A)**; Antibody titers, as determined using SP O **(B)** and SP A **(C)** ELISAs; VN titers for O PA2 **(D)** or A YC **(E)**, as determined using VN test. Data are presented as mean ± SEM of triplicate measurements (*n* = 5/group). Statistical analyses were performed using two-way analysis of variance followed by Tukey’s *post-hoc* test. ^*^ *p* < 0.05; ^**^ *p* < 0.01; ^***^ *p* < 0.001; ^****^ *p* < 0.0001. dpv, days post-vaccination; PC, positive control; NC, negative control; ELISA, enzyme-linked immunosorbent assay; VN, virus neutralization.

To evaluate the induction of humoral immune responses by the test vaccine using D-galacto-D-mannan as an adjuvant, an experiment was performed according to the design shown in **Figure 3A**.

When the test vaccine containing D-galacto-D-mannan was administered to mice in the Exp group, the antibody titers at 7 dpv (*p* < 0.05, SP O ELISA using the VDPro^®^ Kit; *p* < 0.01, SP A ELISA using the PrioCheck™ Kit), 28 dpv (*p* < 0.01, SP O ELISA; *p* < 0.001, SP A ELISA), 56 dpv (*p* < 0.001, both SP O and A ELISA), and 84 dpv (*p* < 0.0001, SP O ELISA; *p* < 0.001, SP A ELISA) were higher than those in the PC group vaccinated without D-galacto-D-mannan. Antibody titers in the NC group did not change (**Figures 3B, C**).

The VN titers were higher in the Exp group administered the vaccine containing D-galacto-D-mannan together with the O PA2 + A YC antigen, 7 dpv (*p* < 0.01, O PA2; *p* < 0.05, A YC), 28 dpv (*p* < 0.05, both O PA2 and A YC), 56 dpv (*p* < 0.001, O PA2; *p* < 0.01, A YC), and 84 dpv (*p* < 0.01, O PA2; *p* < 0.001, A YC), compared with the PC group. VN titers in the NC group did not change (**Figures 3D, E**).

**Figure 4:**
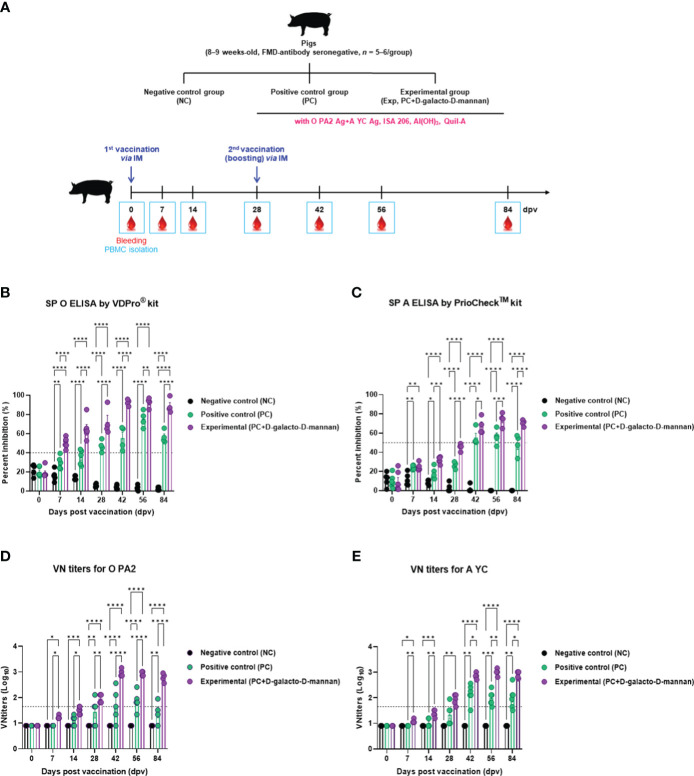
FMD vaccine containing D-galacto-D-mannan elicits early, mid-, and long-term immune responses in pigs. For the pig experiments, FMDV type O and type A antibody-seronegative animals (8–9 weeks old) were used. The pigs were divided into three groups (*n* = 5 or 6/group) and administered inactivated bivalent FMDV vaccine without (PC group) or with 1 mg/dose/pig D-galacto-D-mannan (Exp group). PC group received FMDV type O (O PA2) and type A (A YC) antigens (15 + 15 μg/dose/mL, one dose for cattle and pig use) with ISA 206 (oil-based emulsion, 50%, w/w), 10% aluminum hydroxide, and 150 μg of Quil-A. Vaccination was performed twice at 28-day intervals, with 1 mL of vaccine (one dose) injected *via* the deep intramuscular route into animal necks. The NC group was injected with an equal volume of phosphate-buffered saline. Blood samples were collected 0, 7, 14, 28, 42, 56, and 84 dpv for serological assays. **(A–E)** Experimental strategy **(A)**; antibody titers, as determined using SP O **(B)** and SP A **(C)** ELISAs; VN titers for O PA2 **(D)** or A YC **(E)**, as determined using VN test. Data are presented as mean ± SEM of triplicate measurements (*n* = 5 or 6/group). Statistical analyses were performed using two-way analysis of variance followed by Tukey’s *post-hoc* test. ^*^ *p* < 0.05; ^**^ *p* < 0.01; ^***^ *p* < 0.001; ^****^ *p* < 0.0001. FMD, foot-and-mouth disease; FMDV, foot-and-mouth disease virus; PC, positive control; NC, negative control; dpv, days post-vaccination; ELISA, enzyme linked immunosorbent assay; VN, virus neutralization.

The targeted animal experiments using FMD antibody-seronegative pigs to evaluate the induction of humoral immune response by test vaccine with D-galacto-D-mannan in pigs are shown in **Figure 4A**.

The FMDV type O-specific antibody titers (SP O ELISA using the VDPro^®^ kit) of the Exp group receiving the vaccine containing D-galacto-D-mannan were higher than that of the PC group at each sampling point. In particular, after boosting 28 dpv, the antibody titers of the Exp group were the highest at 42 dpv, and the elevated antibody titers were maintained until 84 dpv. The NC group showed no significant changes (**Figure 4B**).

When the antibody titers were measured using SP A ELISA using the PrioCheck™ kit, it was found to increase somewhat slowly compared with that determined using the SP O ELISA.

However, 7 dpv after the first vaccination, the Exp group antibody titers increased steadily and significantly up to 28 dpv compared with the PC group. After boosting, Exp group antibody titers were seropositive up to 84 dpv, whereas antibody titers in the PC group were seropositive up to 56 dpv. The NC group showed no significant changes (**Figure 4C**).

Similar to the antibody titers measured by SP ELISA, the VN titers were higher in all dpv in the Exp group compared with the PC group (**Figures 4D, E**).

**Figure 5:**
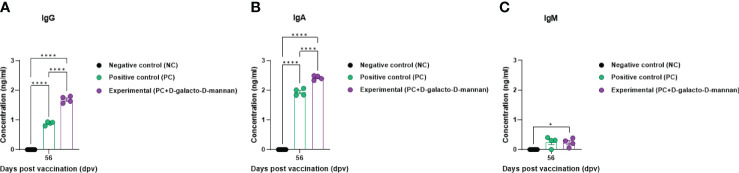
FMD vaccine containing D-galacto-D-mannan elevates levels of immunoglobulin subtypes, such as IgG, IgA, and IgM in pigs. For the pig experiments, FMDV type O and type A antibody-seronegative animals (8–9 weeks old) were used. Pigs were divided into three groups (*n* = 5 or 6/group) and administered inactivated bivalent FMDV vaccine without (PC group) or with (Exp group) 1 mg/dose/pig D-galacto-D-mannan. The PC group received FMDV type O (O PA2) and A (A YC) antigens (15 + 15 μg/dose/mL, one dose for cattle and pig use) with ISA 206 (oil-based emulsion, 50%, w/w), 10% aluminum hydroxide, and 150 μg of Quil-A. Vaccination was performed twice at 28-day intervals with 1 mL of vaccine (one dose) injected *via* a deep intramuscular route into animal necks. The NC group was injected with an equal volume of phosphate-buffered saline. Blood samples were collected 0, 7, 14, 28, 42, 56, and 84 dpv for serological assays. Data are presented as mean ± SEM of triplicate measurements (*n* = 5 or 6/group). Statistical analyses were performed using two-way analysis of variance followed by Tukey’s *post-hoc* test. ^*^ *p* < 0.05; ^**^ *p* < 0.01; ^***^ *p* < 0.001; and ^****^ *p* < 0.0001. **(A–C)** IgG **(A)**; IgA **(B)**; and IgM **(C)** concentrations. FMD, foot-and-mouth disease; FMDV, foot-and-mouth disease virus; PC, positive control; NC, negative control; dpv, days post-vaccination.

The Ig isotype ELISAs performed using serum samples from vaccinated pigs at 56 dpv are shown in **Figure 4A**. The IgG and IgA concentrations were significantly higher in the Exp group than in the control group, whereas no significant difference was observed in the IgM concentration between the Exp and PC groups (**Figures 5A–C**).

Pigs vaccinated with a vaccine containing D-galacto-D-mannan showed no local side effects (granulomas or inflammation) at the injection site compared with the PC group (**Supplementary Figures 5A–C**).

These results demonstrated that the test vaccine containing D-galacto-D-mannan elicited superior early, mid-, and long-term immune responses.

### 3.4 FMD vaccine containing D-galacto-D-mannan exhibits immunomodulatory functions *via* mediating the gene expression of PRRs, transcription factors, cytokines, and costimulatory molecules

**Figure 6:**
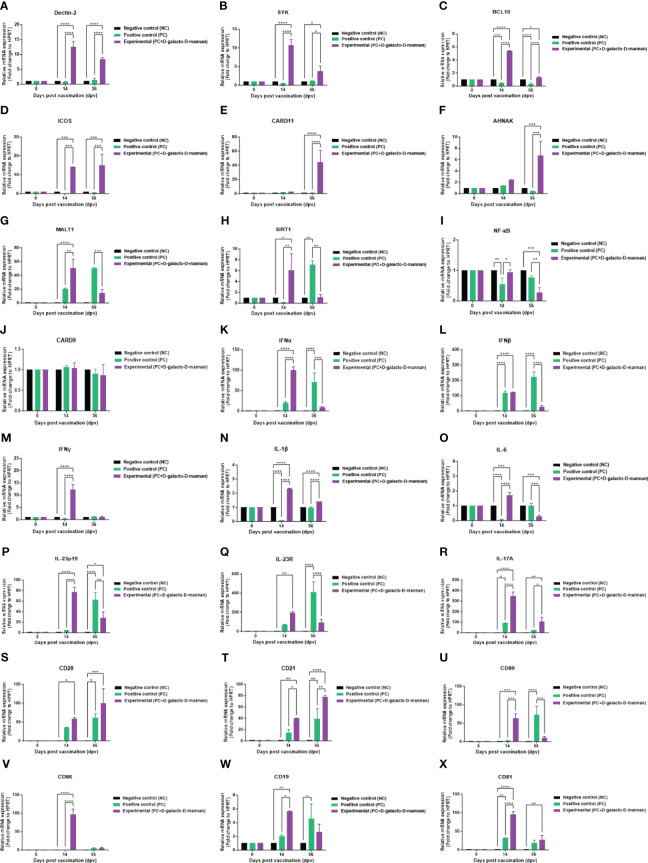
FMD vaccine containing D-galacto-D-mannan induces gene expression of PRRs, transcription factors, cytokines, and costimulatory molecules in porcine PBMCs. Porcine PBMCs isolated from the whole blood of vaccinated pigs (*n* = 5 or 6/group), as described in **Figure 4A**, were used for quantitative real-time polymerase chain reaction. Gene expression levels were normalized to those of HPRT and are presented as relative ratios compared to control levels. **(A–X)** Gene expression levels of Dectin-2 **(A)**; SYK **(B)**; BCL10 **(C)**; ICOS **(D)**; CARD11 **(E)**; AHNAK **(F)**; MALT1 **(G)**; SIRT1 **(H)**; NF-κB **(I)**; CARD9 **(J)**; IFNα **(K)**; IFNβ **(L)**; IFNγ **(M)**; IL-1β **(N)**; IL-6 **(O)**; IL-23p19 **(P)**; IL-23R **(Q)**; IL-17A **(R)**; CD28 **(S)**; CD21 **(T)**; CD80 **(U)**; CD86 **(V)**; CD19 **(W)**; and CD81 **(X)**. Statistical analyses were performed using two-way analysis of variance followed by Tukey’s *post-hoc* test. ^*^ *p* < 0.05; ^**^ *p* < 0.01; ^***^ *p* < 0.001; ^****^ *p* < 0.001. FMD, foot-and-mouth disease; PRRs, pattern-recognition receptors; PBMCs, peripheral blood mononuclear cells.

To investigate the mechanisms underlying the innate and adaptive immune responses elicited by vaccines containing D-galacto-D-mannan, according to the strategy presented in **Figure 4A**, qRT-PCR was performed using porcine PBMCs at 14 and 56 dpv sampling points (**Figures 6A–X**).

The expression of PRR and transcription factors, such as Dectin-2 (**Figure 6A**), SYK (**Figure 6B**), BCL10 (**Figure 6C**), and ICOS (**Figure 6D**), was higher in the Exp group than the control groups (PC and NC) both at 14 and 56 dpv. The expression of CARD11 (**Figure 6E**) and AHNAK (**Figure 6F**) was higher in the Exp than the PC group at 56 dpv. Conversely, the expression of MALT1 (**Figure 6G**), SIRT1 (**Figure 6H**), and NF-κB (**Figure 6I**) was higher in the Exp than the PC group at 14 dpv, but lower at 56 dpv. Finally, the expression of CARD9 (**Figure 6J**) showed no significant difference compared with the PC and NC groups at both 14 and 56 dpv.

The cytokine expression levels in pigs vaccinated with D-galacto-D-mannan were generally higher than those in the PC group 14 dpv but tended to be lower at 56 dpv. IFNα (**Figure 6K**), IFNβ (**Figure 6L**), IFNγ (**Figure 6M**), IL-1β (**Figure 6N**), IL-6 (**Figure 6O**), IL-23p19 (**Figure 6P**), and IL-23R (**Figure 6Q**) expression in the Exp was higher than the PC group 14 dpv but lower at 56 dpv. IL-17A (**Figure 6R**) expression in the Exp was higher than the PC group at both 14 and 56 dpv.

The expression of co-stimulatory molecules, such as CD28 (**Figure 6S**) and CD21 (**Figure 6T**), was higher in the Exp group than the PC group at 14 and 56 dpv. Conversely, CD80 (**Figure 6U**), CD86 (**Figure 6V**), CD19 (**Figure 6W**), and CD81 (**Figure 6X**) expressions were higher in the Exp group than the PC group 14 dpv but lower or showed no difference at 56 dpv. Our findings elucidate the immunological mechanisms induced by D-galacto-D-mannan, suggesting a background for the strong cellular and humoral immune responses induced by FMD vaccines containing D-galacto-D-mannan.

### 3.5 Combining intramuscular vaccination of FMD vaccine with oral administration of D-galacto-D-mannan simultaneously induces systemic and mucosal immunity and elicits long-lasting immune responses

To evaluate the effect of simultaneous induction of systemic immunity and mucosal immunity, and long-lasting immune response through a combined program of intramuscular vaccination of FMD vaccine and oral administration of D-galacto-D-mannan, an experiment was performed according to the strategy in **Supplementary Figure 6A**. When intramuscular single-dose vaccination of FMD vaccine was combined with oral administration of D-galacto-D-mannan for 28 days, the immune response using SP O ELISA and VN test lasted until 84 dpv (**Supplementary Figure 6B, C**).

## 4 Discussion

To date, various approaches have been proposed to develop novel FMD vaccines; however, the shortcomings of commercialized vaccines, such as (1) slow antibody titer induction, (2) difficulty in initial host defense, (3) short antibody titer persistence, (4) periodic and repeated vaccinations, and (5) side effects at the vaccination site, have not been addressed (33, 34). Therefore, we developed a novel FMD vaccine containing D-galacto-D-mannan as an adjuvant (immunostimulant).

We have previously confirmed that furfurman, a Dectin-2 agonist, promoted porcine PBMC proliferation; when added as an adjuvant to a vaccine, it simultaneously induced cellular and humoral immunity in mice and pigs, the experimental and target animals, respectively (18). We also evaluated host defense against FMDV O and FMDV A challenge in pigs vaccinated with the FMD bivalent (O+A) vaccine containing furfurman as an adjuvant. The FMD vaccine containing furfurman completely protected the host against heterologous FMDV infection (data not shown).

Furfurman is a cell wall component derived from *Malassezia furfur*, an opportunistic skin fungal pathogen recognized by C-type lectin receptors, especially Dectin-2 (35). Its receptors play a pivotal role in antifungal innate immune response. Dectin-2 binds mannose carbohydrates, such as the mannose-coated lipoarabinomannan of mycobacteria (36) and α-mannan found in *Candida albicans* (37). Upon binding, Dectin-2 associates with the Fc receptor gamma chain (38) and signals *via* SYK and CARD9/BCL-10/MALT1 (CBM complex), triggering NF-κB activation and subsequent proinflammatory cytokine production (39). Despite the importance of furfurman in inducing immune responses, safety issues related to its origin in pigs remains a concern since the fungus could affect animals. Moreover, considering veterinary vaccine expenses, the cost of furfurman is high, complicating its application to FMD vaccines. Therefore, we established a strategy to use a furfurman replacement as an adjuvant with guaranteed safety and economic efficiency; hence, D-galacto-D-mannan extracted from the cell wall of *Ceratonia siliqua* was used in this study.

Prior to *in vitro* studies, D-galacto-D-mannan-mediated cytotoxicity was assessed. Since cytotoxicity was not observed at concentrations of 0–5 μg/mL (**Supplementary Figure 1**), subsequent experiments were performed assuming that treatment with D-galacto-D-mannan in this concentration range was safe. To evaluate D-galacto-D-mannan-mediated innate and adaptive (cellular) immune response, we quantified D-galacto-D-mannan-mediated IFNγ secretion using the ELISpot assay with murine PECs and porcine PBMCs, and confirmed that D-galacto-D-mannan exerted a promising adjuvant effect when co-treated with the FMD viral antigen (**Figure 1**). Both PECs and PBMCs contain immune cells closely related to innate and adaptive immune responses, including DCs, MΦs, T cells, B cells, and unconventional T cells [gamma delta T (γδ T), invariant natural killer T (iNKT), and mucosal-associated invariant T (MAIT) cells], making them suitable for evaluating cellular immune responses (18). IFNγ is mainly secreted by T, NK, NKT, antigen-presenting cells (MΦs and DCs), and B cells. In addition, IFNγ secreted by antigen presenting and NK cells is closely related to early host defense (40, 41). Oh et al. (42) reported that IFNγ secretion could be restimulated in vaccinated cows that displayed high VN titer levels on the day of challenge, indicating a direct correlation between FMD vaccine-induced protection and VN antibodies and IFNγ. Therefore, D-galacto-D-mannan has the potential to elicit strong host defense through an enhanced innate immune response and adaptive (cellular and humoral) immune response. The results in sections 3.2 (**Supplementary Figure 2**, **Figure 2**) and 3.3 (**Figures 3**, **4**) demonstrated that D-galacto-D-mannan-mediated cellular immune responses promoted rapid increase in antibody and VN titers to host-defensible levels (3). To evaluate vaccine efficacy, long-term immunity is an important indicator to consider in addition to initial protection against viral infection. This study focused on the induction of not only early and mid-term immunity but also long-term immunity by inducing cellular and humoral immune responses of the FMD vaccine containing D-galacto-D-mannan.

The antibody and VN titers of the Exp group vaccinated with FMD vaccine containing D-galacto-D-mannan were superior to those of the control (PC and NC) groups in all aspects, including rate of increase, level, and maintenance. The Exp group showed significantly higher antibody and VN titers at 7, 28, 56, and 84 dpv compared with the control group. Even at 7 dpv, complete host protection was expected at 28, 56, and 84 dpv, provided that the challenged mice induced host protection against FMDV infection. These results demonstrate that the FMD vaccine containing D-galacto-D-mannan can induce early, mid-term, and long-term immunity after vaccination, leading to a rapid, long-lasting, and potent host defense.

Antibodies closely related to the humoral immune response are secreted by B cells. B cells produced in the bone marrow undergo random genetic recombination to produce antibodies that specifically bind to different antigens. Among others, VN antibodies are part of the adaptive (humoral) immune response that can fight and eliminate viral and microbial toxins. Unlike non-neutralizing antibodies, VN antibodies bind specifically to viral antigens and prevent viruses from infecting and destroying host cells (43, 44). Therefore, VN titers are an indirect indicator of host defense. VN titers >1.74 (Log_10_) have been reported to elicit host defense by the FMD vaccine (45). In addition, according to Korea’s FMD vaccine efficacy validation guidelines, host defense is induced when the VN titers are >1.65 (Log_10_) (7). Based on our previous study showing complete host protection against FMDV infection when VN titers > 1.65 (Log_10_), the Exp group in this study will also induce host protection against FMDV infection from 28 to 84 dpv (29). The VN titers of the group vaccinated with FMD vaccine with D-galacto-D-mannan was maintained above 2 (Log_10_) for a long duration following the second vaccination; hence, this novel FMD vaccine can induce long-term host protection.

Ig isotype ELISA confirmed the IgG, IgA, and IgM levels specifically induced by the bivalent (O PA2+A YC) FMD vaccine containing D-galacto-D-mannan, demonstrating that D-galacto-D-mannan significantly contributes to the host humoral immune response (**Figure 5**). Of the five isotypes (IgG, IgA, IgM, IgD, and IgE) found in mammals, IgG plays a critical role in humoral immunity, especially in the induction of neutralizing antibodies. IgG is classified into diverse subsets with their relative abundance, size, complement activation, immune complex formation, binding to Fc receptors, and effector functions (46, 47). IgA is detected in the serum and intestinal mucosa and mostly secreted by B-cell responses in the gut (48–50). IgA is also secreted by T-cell-independent and dependent pathways and based on the pathways, and has different functions (51). IgA produced by the T-cell-independent pathway targets non-invasive commensals, whereas IgA produced by the T-cell-dependent pathway coats penetrant commensals and invasive pathogens (52–55). Thus, the enhanced humoral immune response induced by FMD vaccine with D-galacto-D-mannan significantly increases IgG and IgA levels, enhancing systemic immunity and consequently contributing to long-term immunity.

In our previous study, similar to the qRT-PCR results of the Exp group including the Dectin-1 agonist, we assumed that the gene expression levels of downstream signals of the Dectin-2-mediated signaling pathway would be high for the Exp group including the Dectin-2 agonist (23, 29, 37). However, the results showed low expression levels of specific transcription factors (CARD9 and NF-κB) and several inflammatory cytokines (IL-1β and IL-6) in contrast to high expression levels of CARD11 and SIRT1. This maintains homeostasis within the host by suppressing excessive inflammatory immune responses.

Herein, D-galacto-D-mannan, used as a Dectin-2 agonist, is extracted from plant cell walls; Dectin-2 is essential for host protection against *Candida albicans via* Th17 cell response (24, 37). Previous studies have reported that D-galacto-D-mannan exhibits antioxidant activity (28), which, along with its anti-inflammatory activities, is associated with increased SIRT1 expression levels (56). In the Exp group, cytokines (IL-1β, IL-6, IL-23p19, and IL-17A) with higher expression at 14 than 56 dpv are essential for maturation and differentiation of cells involved in innate and humoral (cellular) immunity. IL-23 (IL-12p40/IL-23p19) contributes to the early immune response by stimulating Th17, γδ T, iNKT, and innate lymphoid cells (57–59). IL-23p19 also induces IFNγ secretion in Th17 cells, eliciting cellular and humoral immune responses (41, 60). IL-1β and IL-6 promote differentiation of Th17 cells. IL-1β enhances the metabolic fitness of rapidly dividing Th17 cells during an inflammatory response by inducing phosphorylation of the mammalian target of rapamycin (mTOR) in Th17 cells (61). IL-6 induces IL-17A and IL-23R by promoting Th17 cell-related gene expression (62, 63). Expression levels of genes involved in the adaptive immune response increased at 56 dpv compared with 14 dpv.

Increased CARD11, ICOS, AHNAK, CD28, and CD21 expression levels demonstrate enhanced adaptive immune response by promoting maturation and differentiation of T and B cells. First, as CARD11 is mainly activated by CD28, T-cell receptor co-stimulation, and B-cell receptor signaling to form the CBM complex, the increased CARD11 expression in long-term immunity demonstrates that T and B cells are significantly stimulated (64, 65). ICOS, a member of the B7 family that binds to CD28, can be derived from T cells during the immune response and exhibits high expression, especially on follicular T helper cells (66–68). Yamasaki et al. (36) reported that Dectin-2/SYK signaling induced IL-2 secretion upon nuclear factor of activated T cells (NFAT) and Ca^2+^ influx stimulation. AHNAK is also involved in Ca^2+^ influx/NFAT signaling and induces IL-2 and IFNγ secretion *via* CD4+ T and CD8+ T-cell activation (69, 70). CD28 and CD21 are involved in T-cell and B-cell activation, respectively, and are important costimulatory molecules in inducing adaptive immune responses (71). In the PC group, the expression of cytokines and costimulatory molecules 56 dpv after the second vaccination were enhanced compared with that observed at 14 dpv, whereas in the Exp group, the cytokine and costimulatory molecule expression was higher at 14 than 56 dpv after the first vaccination (**Figure 6**). These results provide evidence that the Exp group administered the FMD vaccine containing D-galacto-D-mannan induced a significantly faster and stronger cellular immune response than the control (PC, NC) groups, thereby effectively inducing humoral immune responses such as antibody titers and VN titers.

In addition to the well-known Dectin-2-related mechanism, we proposed novel findings in this study, including immunostimulatory mechanisms triggered by plant-derived Dectin-2 agonist, as well as immunoregulatory mechanisms of the host immune response through suppression of excessive inflammatory responses. However, repeated large-scale blood collection may cause side effects, such as hemorrhagic shock, and adversely impact normal growth and the formation of vaccine-mediated immune responses in animals. For Western blot, very few types of pig antibodies are commercially available, making it difficult to identify other potential mechanisms. Moreover, commercially available porcine primary antibodies for Western blot and cytokine ELISA kits are extremely limited, which hinders the complete assessment of the diverse immune-related mechanisms. Therefore, to better understand and elucidate the diverse and overall mechanism of the FMD vaccine containing D-galacto-D-mannan, we presented gene expression levels through qRT-PCR instead of quantifying protein expression levels related to immune response with Western blot or ELISA. Based on this study, we intend to identify various mechanisms caused by FMD vaccines containing D-galacto-D-mannan through a systemic approach, such as RNA sequencing, in future studies.

From the results in **Figure 6**, it can be observed that the expression of cytokine-related genes such as IL-1β, IL-23p19, and IL-17A, which play an important role in the induction mechanism of mucosal immunity, was significantly increased. Therefore, the effect of inducing an immune response by D-galacto-D-mannan was evaluated using vaccination routes that can induce mucosal immunity, such as oral administration in addition to intramuscular injection. A long-lasting immune response was demonstrated when D-galacto-D-mannan was administered orally daily for 28 days along with a single dose of control vaccine (**Supplementary Figure 6**). Based on these results, it was confirmed that D-galacto-D-mannan is effective in inducing not only systemic immunity but also mucosal immunity, and we plan to confirm the effect of inducing mucosal immune responses through various administration routes such as nasal administration in the future.

However, histological analysis was not performed to provide objective evidence regarding the D-galacto-D-mannan-mediated cellular response and subsequent viral clearance *via* challenge studies, which is a limitation of this study. Therefore, in the next study, we plan to conduct expanded animal research targeting more animals and present these results.

Our study provides novel perspectives for establishing vaccine formulas and vaccination strategies for FMD and other difficult-to-prevent and control viral diseases.

## Data availability statement

The original contributions presented in the study are included in the article/**Supplementary Material**. Further inquiries can be directed to the corresponding authors.

## Ethics statement

The animal study was approved by Animal and Plant Quarantine Agency (APQA) Ethics Committee (Certification No.: IACUC-2022-670 and 2023-753). The study was conducted in accordance with the local legislation and institutional requirements.

## Author contributions

HWK: Formal Analysis, Investigation, Software, Validation, Visualization, Writing – original draft, Writing – review & editing. M-KK: Investigation, Writing – original draft. SHP: Investigation, Writing – original draft. SS: Investigation, Writing – original draft. GSK: Investigation, Writing – original draft. DYK: Investigation, Writing – original draft. J-HP: Resources, Writing – review & editing. S-MK: Resources, Writing – review & editing. J-SL: Supervision, Writing – review & editing. MJL: Conceptualization, Formal Analysis, Funding acquisition, Investigation, Methodology, Project administration, Resources, Software, Supervision, Validation, Visualization, Writing – original draft, Writing – review & editing.
