# Supplementary material for: D-galacto-D-mannan-mediated Dectin-2 activation orchestrates potent cellular and humoral immunity as a viral vaccine adjuvant
Source: Front Immunol. 2024 Feb 16;15:1330677. doi: 10.3389/fimmu.2024.1330677 (PMC10904532; doi:10.3389/fimmu.2024.1330677)
Supplement: Supplementary file 1 [file DataSheet_1.pdf]

Supplementary Material

D-galacto-D-mannan mediated Dectin-2 activation orchestrates potent cellular and humoral immunity as a viral vaccine adjuvant

Hyeong Won Kim[1], Mi-Kyeong Ko[1], So Hui Park[1], Seokwon Shin[1], Gang Sik Kim[1], Dong Yun Kwak[1], Jong-Hyeon Park[1], Su-Mi Kim[1], Jong-Soo Lee[2*], Min Ja Lee[1*]

[1] Center for Foot-and-Mouth Disease Vaccine Research, Animal and Plant Quarantine Agency, Gimcheon-si, Gyeongsangbuk-do, Republic of Korea

[2]College of Veterinary Medicine, Chungnam National University, Daejeon, Republic of Korea

*** Correspondence:**
Jong-Soo Lee[2]; Min Ja Lee[1]
jongsool@cnu.ac.kr (J.-S.L.); herb12@korea.kr (M.J.L.)

1 Supplementary Table and Figures

1.1 Supplementary Table

Supplementary Table 1. List of primer sequences for qRT-PCR.

Target	Forward/Reverse	Sequence (5'- 3')	Length (mer)
hDectin-2	hDectin-2 F	GCTGAGTCTCTGGGCAACAT	20
	hDectin-2 R	TGAGGTTGCTGCTCTTGCAT	20
SYK	SYK F	CCAACCACTTGCCCTTCTTC	20
	SYK R	ATGGTGTAGTGATGCGCCTT	20
CARD9	CARD9 F	CCGCAGCTCTACAAGAAGGT	20
	CARD9 R	TCTGCAGCTTCATCACCTCG	20
hCARD11	CARD11 F	TGAACGAGGTCATCAAGCTG	20
	CARD11 R	AGCGTCAGCTGCTTCTTCTC	20
BCL10	BCL10 F	ATGGAGCCCGCCGCGCCGTC	20
	BCL10 R	GCTATGATTTTTTCACACAG	20
MALT1	MALT1 F	GTTGGAAGCCCCATTCCACA	20
	MALT1 R	ACTCCACTGCCTCATCTGTTC	21
NF-κB	NF-κB F	TCGCTGCCAAAGAAGGACAT	20
	NF-κB R	AGCGTTCAGACCTTCACCGT	20
ICOS	ICOS F	GGATGTGCAGCCTTTGTTGT	20
	ICOS R	CAGAGCGTACCAAATTGCGG	20
AHNAK	AHNAK F	CACCATCACCGTGACTCGAA	20
	AHNAK R	AGTTCGTGCCGTGGAATCTT	20
IFNα	IFNα F	CATCTGCTCTCTGGGCTGTG	20
	IFNα R	TGAGGGGATCCAAAGTCCCT	20
IFNβ	IFNβ F	TGCAACCACCACAATTCCAGA	21
	IFNβ R	GGTTTCATTCCAGCCAGTGC	20
IFNγ	IFNγ F	GCCATTCAAAGGAGCATGGAT	21
	IFNγ R	CTGATGGCTTTGCGCTGGAT	20

Supplementary Table 1 (*continued*)

Target	Forward/Reverse	Sequence (5'- 3')	Length (mer)
IL-1β	IL-1β F	AGCCAGTCTTCATTGTTCAGGT	22
	IL-1β R	TCATCTCTTTGGGGCCATCAG	21
IL-6	IL-6 F	CTGCAGTCACAGAACGAGTG	20
	IL-6 R	CGGCATCAATCTCAGGTGCC	20
IL-23p19	IL-23p19 F	CCATATCCAGTGCGGGGATG	20
	IL-23p19 R	AGGCCTTGGTGGATCCTTTG	20
IL-23R	IL-23R F	TCCCTCATTGCAAAGCACAA	20
	IL-23R R	GCATCTCCTCTTGCAAGCAAAT	22
IL-17A	IL-17A F	CTCGTGAAGGCGGGAATCAT	20
	IL-17A R	GGTGTGCTCCGGTTCAAGAT	20
CD28	CD28 F	TCAAAGGAGTTCCGGGCATC	20
	CD28 R	CTGAAGCAGGCGGGAGTAAT	20
CD80	CD80 F	TCAGACACCCAGGTACACCA	20
	CD80 R	GACACATGGCTTCTGCTTGA	20
CD86	CD86 F	TTTGGCAGGACCAGGATAAC	20
	CD86 R	GCCCTTGTCCTTGATTTGAA	20
CD21	CD21 F	TGCCATGCCTACAAAGCTGA	20
	CD21 R	GTAGTAACCAGGGCGGCATT	20
CD19	CD19 F	GGACGACAGACTTCCTGAGC	20
	CD19 R	GTTCTGGCCCATCAGGATTA	20
CD81	CD81 F	TCAACAAGGACCAGATCGCC	20
	CD81 R	GAGCGTCTCGTGGAAAGTCT	20
HPRT	HPRT F	CCCAGCGTCGTGATTAGTGA	20
	HPRT R	GCCGTTCAGTCCTGTCCATA	20

1.2 Supplementary Figures

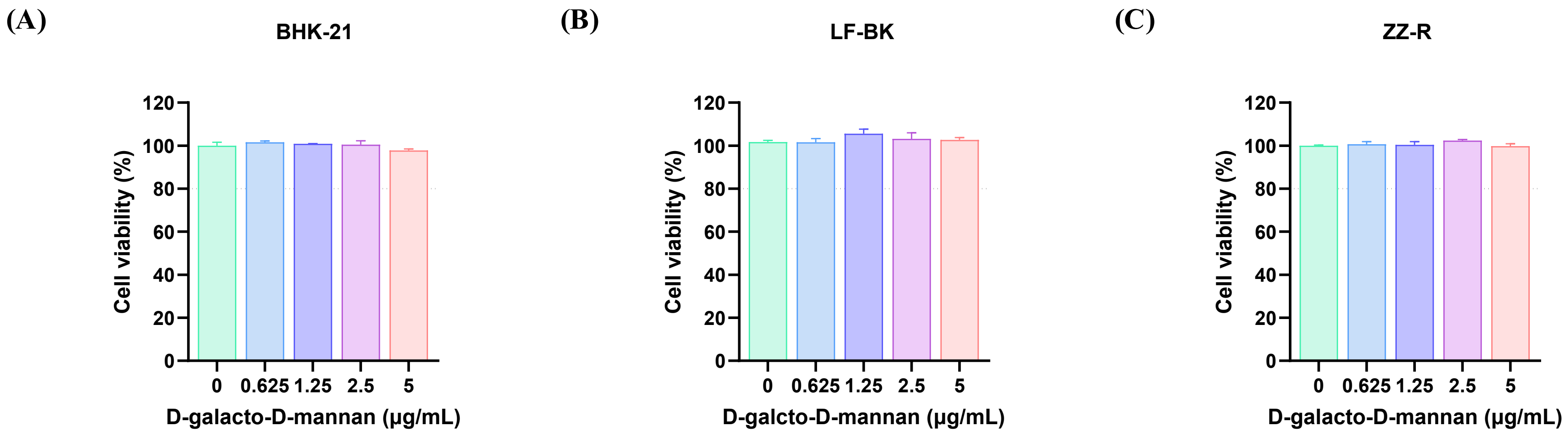

(D)

(E)

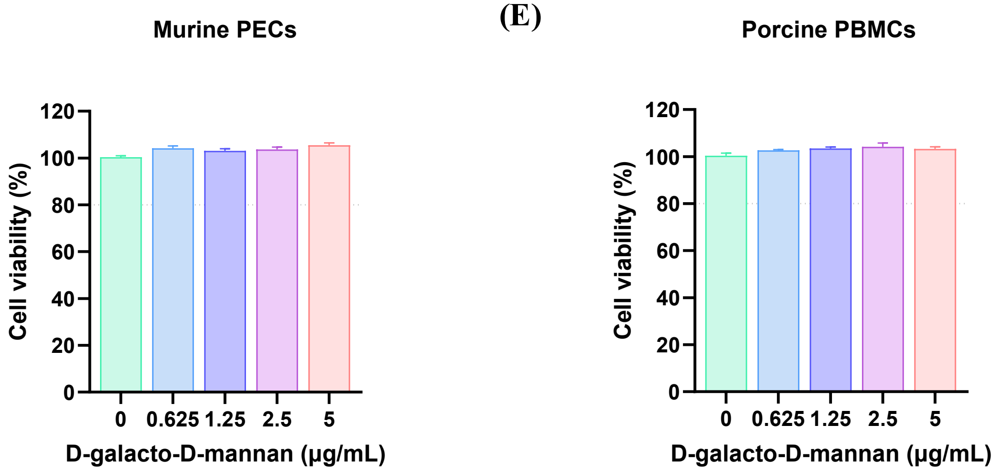

Supplementary Figure 1. Cytotoxicity of D-galacto-D-mannan measured by cell viability assay in BHK-21, LF-BK, ZZ-R, murine PECs and porcine PBMCs. (**A–E**) Cell viability of BHK-21 (**A**); LF-BK (**B**), ZZ-R cells (**C**); murine PECs (**D**); and porcine PBMCs (**E**). Data have been represented as the mean ± SEM of triplicate measurements (n = 3/group). Statistical analyses were performed using one-way ANOVA with Dunnett's *post hoc* test.

(A) Murine PECs

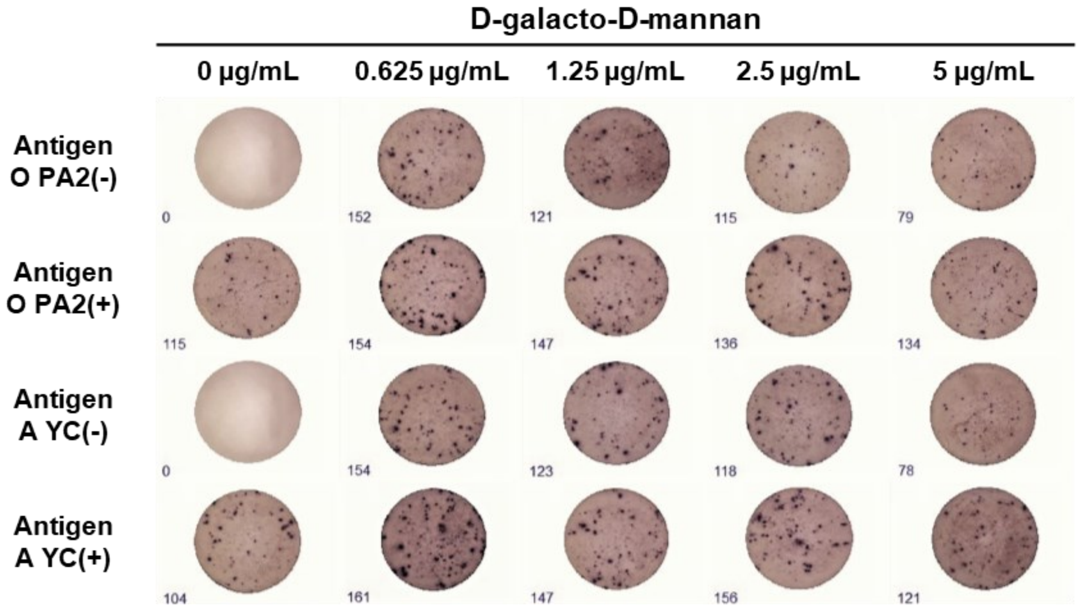

(B) Porcine PBMCs

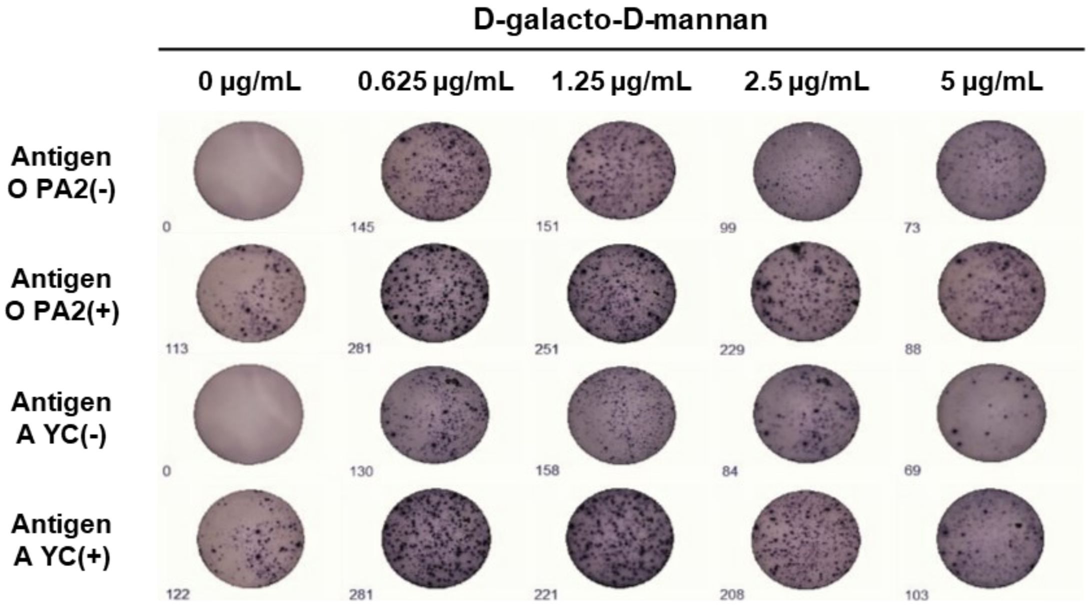

Supplementary Figure 2. IFNγ-secreting cell spots in Figure 1.

IFNγ secretion mediated by O PA2 or A YC antigen, with or without D-galacto-D-mannan, was evaluated using ELISpot assay in murine PECs and porcine PBMCs. Data are presented as spot-forming cells per number of cells in the well, and mean ± SEM of triplicate measurements (*n* = 3/group). (**A, B**) IFNγ-secreting cell spots in murine PECs (**A**); and porcine PBMCs (**B**).

(A)

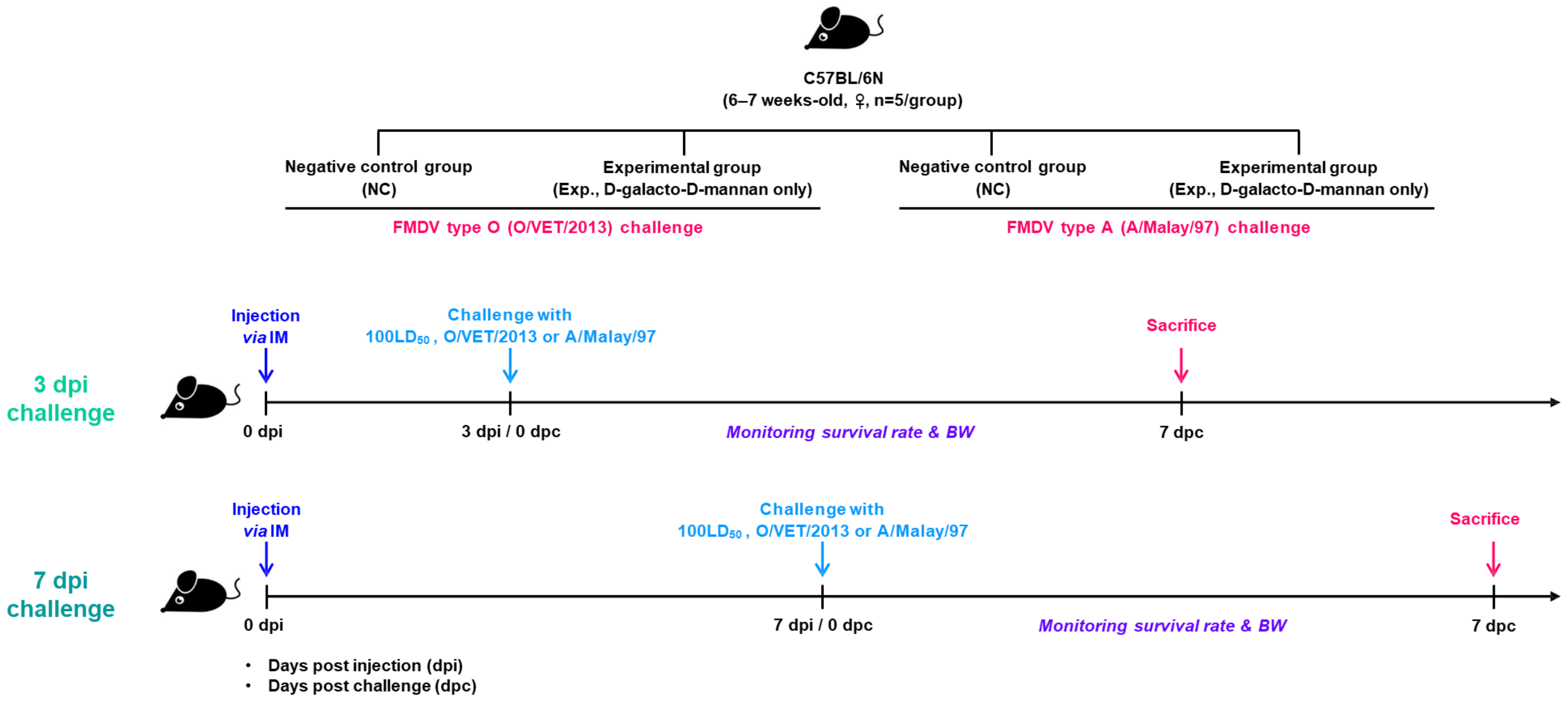
C57BL/6N
(6–7 weeks-old, ♀, n=5/group)
Negative control group (NC)
Experimental group (Exp., D-galacto-D-mannan only)
Negative control group (NC)
Experimental group (Exp., D-galacto-D-mannan only)
FMDV type O (O/VET/2013) challenge
FMDV type A (A/Malay/97) challenge
3 dpi challenge
Injection via IM
Challenge with 100LD50 , O/VET/2013 or A/Malay/97
Sacrifice
0 dpi
3 dpi / 0 dpc
Monitoring survival rate & BW
7 dpc
7 dpi challenge
Injection via IM
Challenge with 100LD50 , O/VET/2013 or A/Malay/97
Sacrifice
0 dpi
7 dpi / 0 dpc
Monitoring survival rate & BW
7 dpc
Days post injection (dpi)
Days post challenge (dpc)

(B)

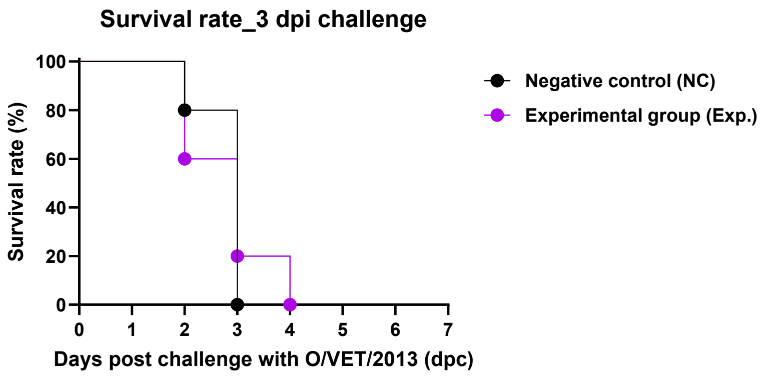
Survival rate_3 dpi challenge
Survival rate (%)
100
80
60
40
20
0
0 1 2 3 4 5 6 7
Days post challenge with O/VET/2013 (dpc)
Negative control (NC)
Experimental group (Exp.)

(C)

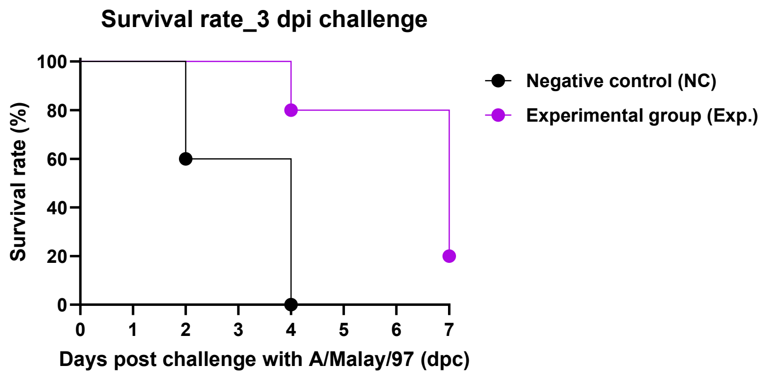
Survival rate_3 dpi challenge
Survival rate (%)
100
80
60
40
20
0
0 1 2 3 4 5 6 7
Days post challenge with A/Malay/97 (dpc)
Negative control (NC)
Experimental group (Exp.)

(D)

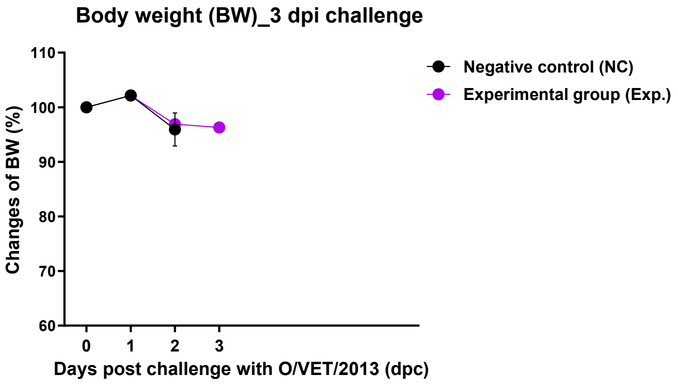
Body weight (BW)_3 dpi challenge
Changes of BW (%)
110
100
90
80
70
60
0 1 2 3
Days post challenge with O/VET/2013 (dpc)
Negative control (NC)
Experimental group (Exp.)

(E)

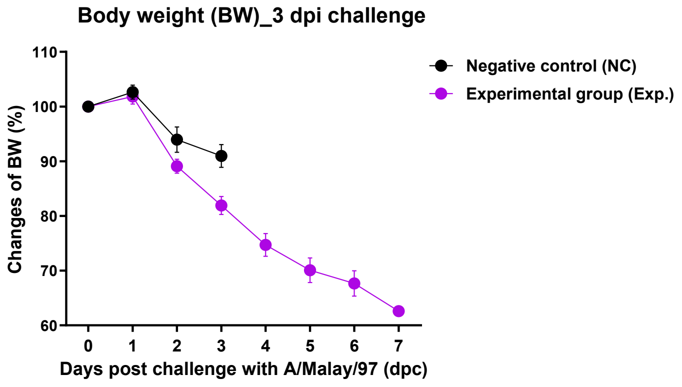
Body weight (BW)_3 dpi challenge
Changes of BW (%)
110
100
90
80
70
60
0 1 2 3 4 5 6 7
Days post challenge with A/Malay/97 (dpc)
Negative control (NC)
Experimental group (Exp.)

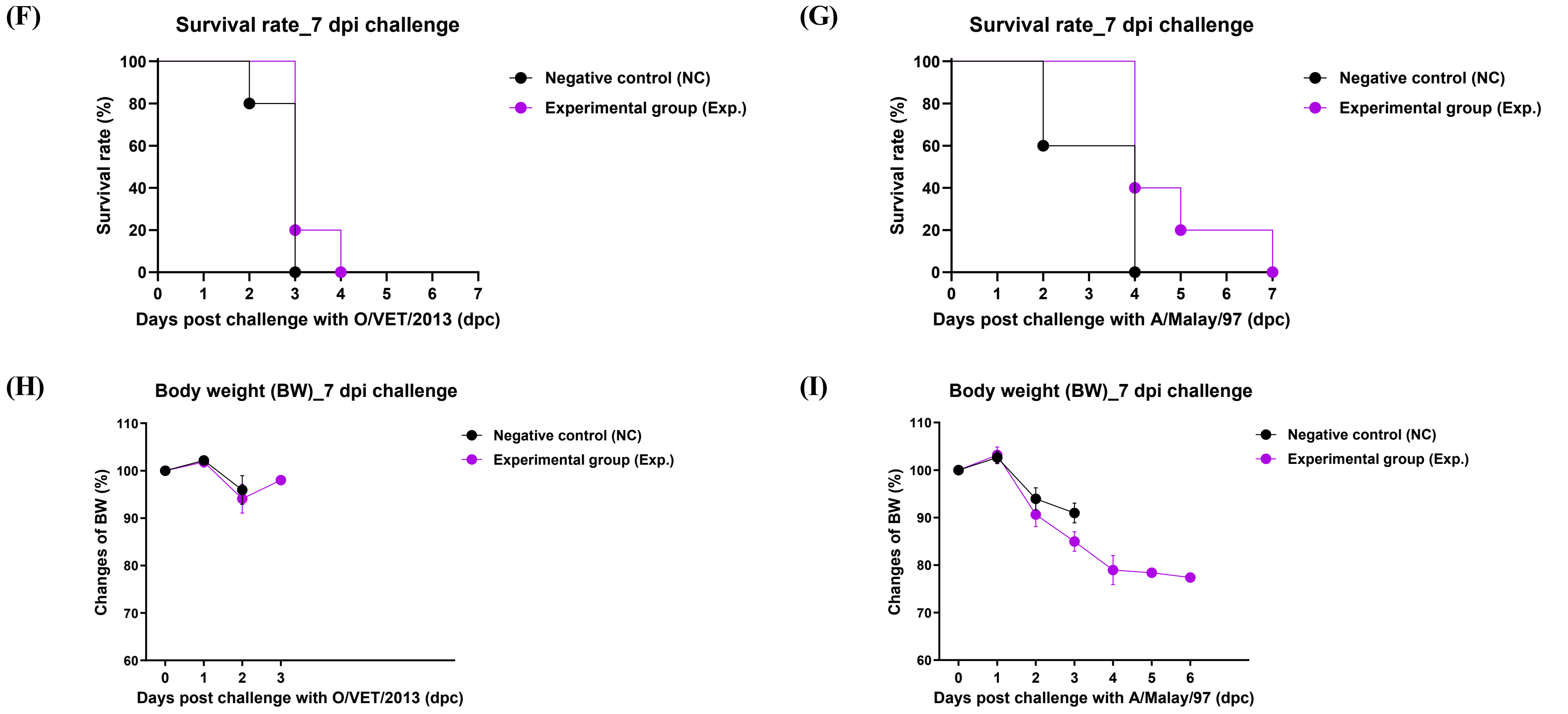

Supplementary Figure 3. D-galacto-D-mannan alone-mediated host defense in early stage of FMDV infection on mice.

C57BL/6 mice (6–7 weeks-old, *n* = 5/group) were administered intramuscularly a D-galacto-D-mannan alone. Mice were challenged with FMDV O (100 LD_{50} O/VET/2013) or FMDV A (100 LD_{50} A/Malay/97) at 3 or 7 days post-injection (dpi) using an intraperitoneal injection. Survival rates and body weights were monitored for 7 days post-challenge (dpc) with the respective viruses. (**A–E**) experimental workflow (**A**); survival rates in 3 dpi challenged group with O/VET/2013 (**B**) or A/Malay/97 (**C**); changes in body weight 3 dpi challenged group with O/VET/2013 (**D**) or A/Malay/97 (**E**); survival rates in 7 dpi challenged group with O/VET/2013 (**F**) or A/Malay/97 (**G**); and changes in body weight 7 dpi challenged group with O/VET/2013 (**H**) or A/Malay/97 (**I**). Data are presented as mean ± SEM of triplicate measurements (*n* = 5/group).

(A)

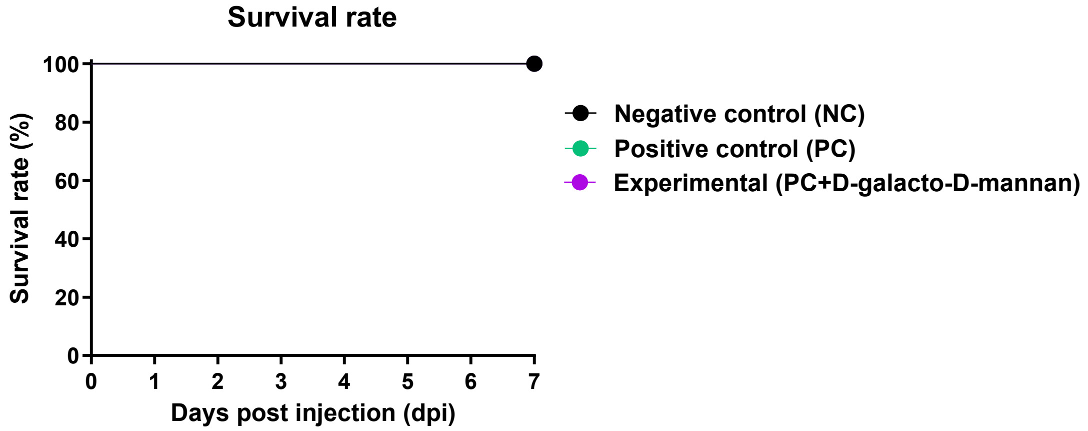

(B)

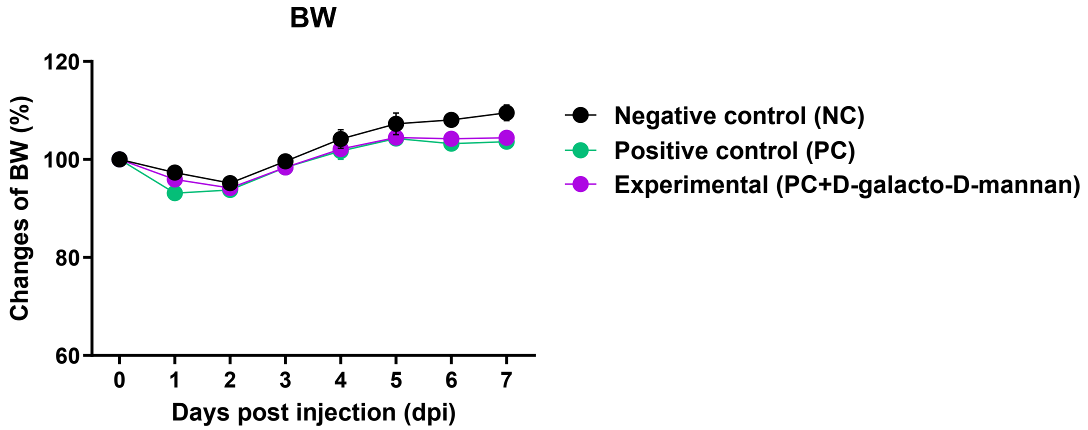

Supplementary Figure 4. Safety of FMD vaccine containing D-galacto-D-mannan as an adjuvant in mice. To test the safety of the FMD vaccine containing D-galacto-D-mannan, mice were administered a vaccine equivalent to 5 times (500 µL) the dose (100 µL) of 1/10 of the target animal (cow or pig) *via* intraperitoneal (IP) injection into the mouse peritoneal cavity, and the survival rate and change of body weight was monitored up to for 7 days post-injection (dpi). (**A, B**) Survival rate (**A**); and change of body weight (**B**).

(A) NC

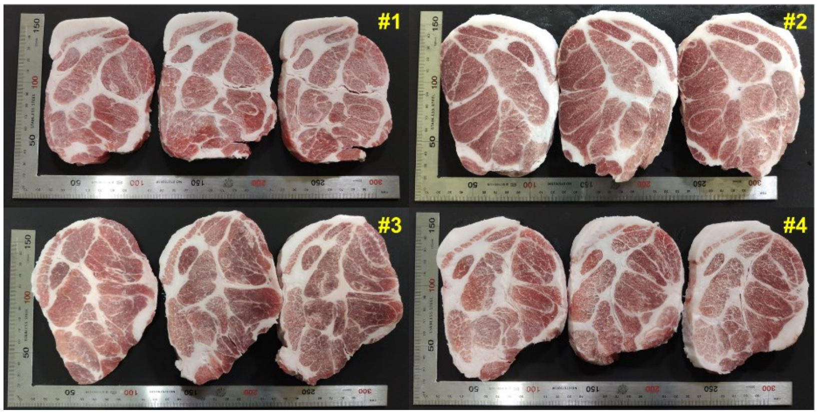

(B) PC

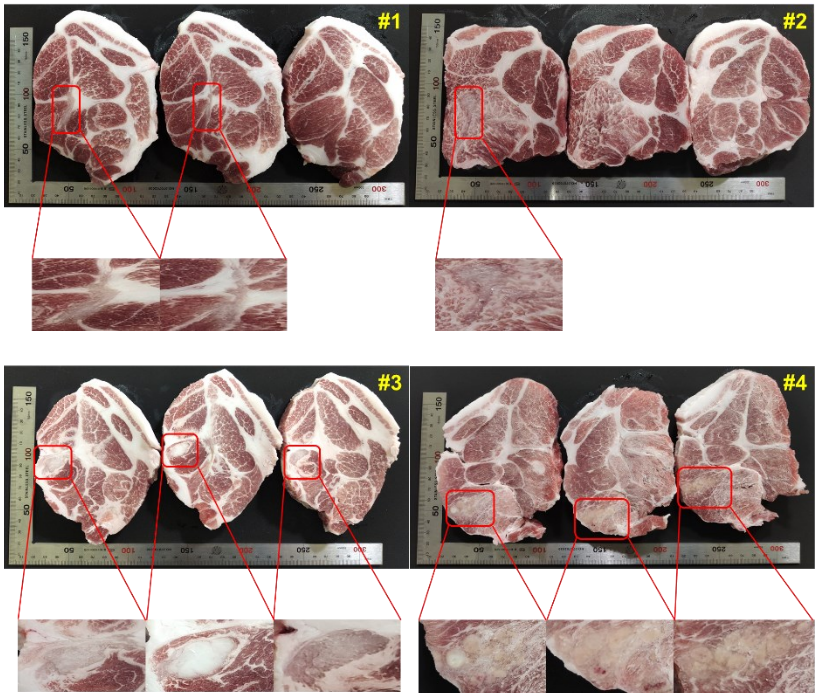

(C) Exp.

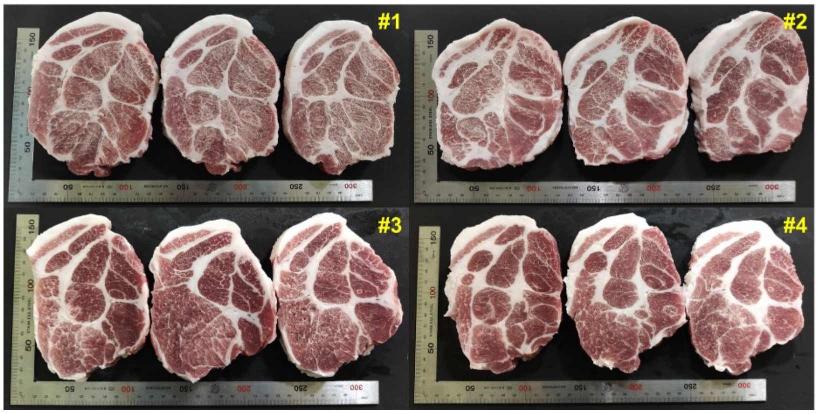

Supplementary Figure 5. Effect of ameliorating FMD vaccine containing D-galacto-D-mannan as an adjuvant-mediated side effects at the vaccination site in pigs.

For the pig experiments, FMDV type O and type A antibody-seronegative animals (8–9 weeks old) were used. The pigs were divided into three groups and administered inactivated bivalent FMDV vaccine without (PC group) or with 1 mg/dose/pig D-galacto-D-mannan (Exp group). PC group received FMDV type O (O PA2) and type A (A YC) antigens (15 + 15 μg/dose/mL, one dose for cattle and pig use) with ISA 206 (oil-based emulsion, 50%, w/w), 10% aluminum hydroxide, and 150 μg Quil-A. Vaccination was performed twice at 28-day intervals, with 1 mL vaccine (one dose) injected via the deep intramuscular route into animal necks. NC group was injected with an equal volume of phosphate-buffered saline. After the end of the experiment, the left and right neck regions of vaccinated pigs (n = 4/group) were resected to observe the formation of a local response to vaccination. (**A–E**) The formation of local response in NC (**A**); PC (**B**); and Exp. group (**C**). Red round square represents the local side effect at the vaccination site.

(A)

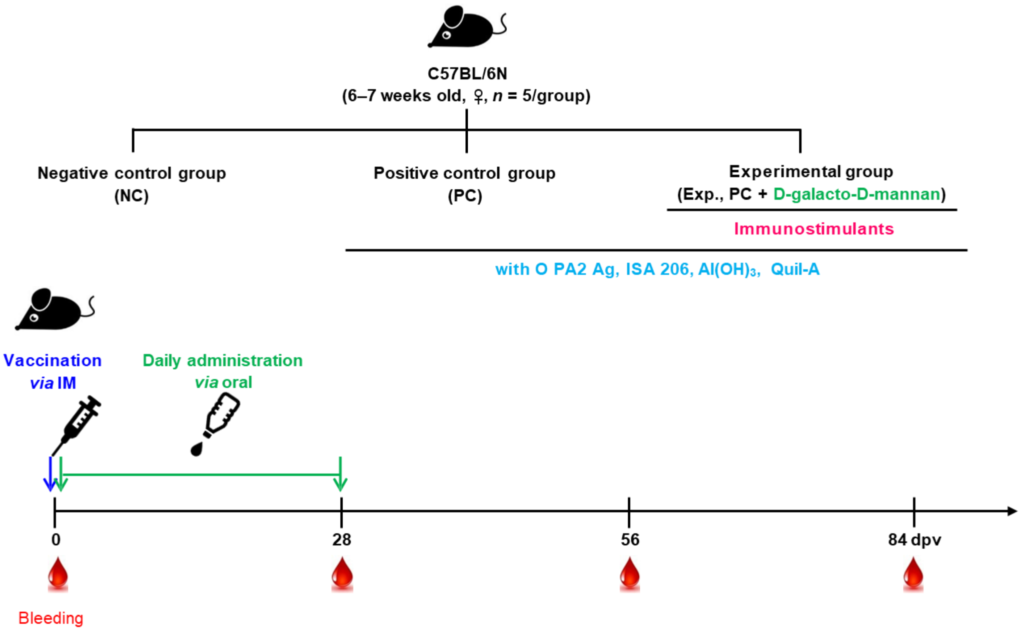

C57BL/6N
(6–7 weeks old, ♀, n = 5/group)
Negative control group
(NC)
Positive control group
(PC)
Experimental group
(Exp., PC + D-galacto-D-mannan)
Immunostimulants
with O PA2 Ag, ISA 206, Al(OH)3, Quil-A
Vaccination
via IM
Daily administration
via oral
0
28
56
84 dpv
Bleeding

(B)

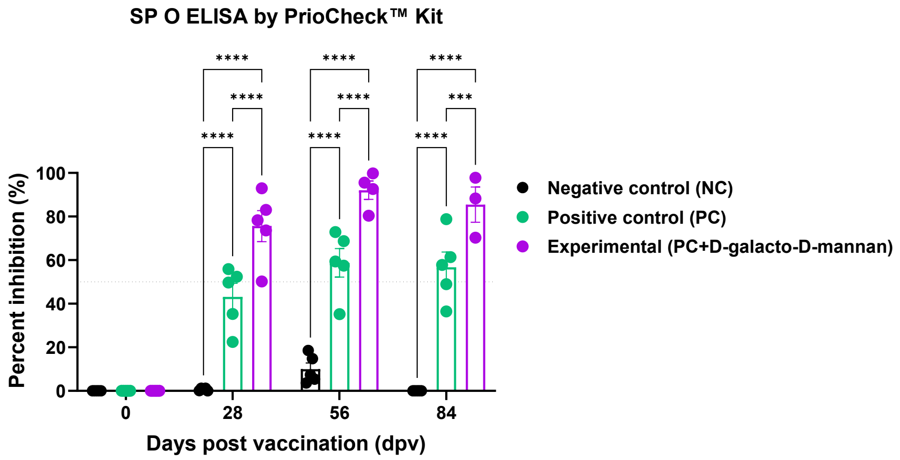

SP O ELISA by PrioCheck™ Kit
Percent inhibition (%)
Days post vaccination (dpv)
Negative control (NC)
Positive control (PC)
Experimental (PC+D-galacto-D-mannan)

(C)

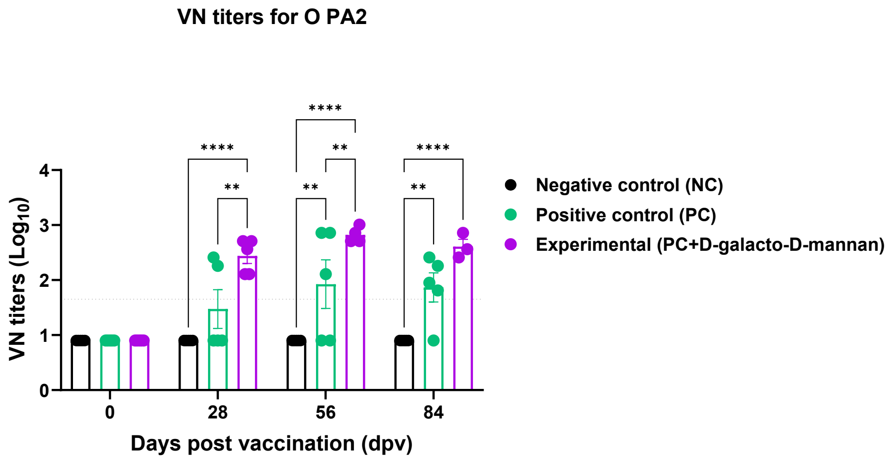

VN titers for O PA2
VN titers (Log10)
Days post vaccination (dpv)
Negative control (NC)
Positive control (PC)
Experimental (PC+D-galacto-D-mannan)

Supplementary Figure 6. Combination of intramuscular vaccination of FMD vaccine and oral administration of D-galacto-D-mannan simultaneously induces systemic and mucosal immunity and elicits long-lasting immune responses.

To verify the effect of simultaneously inducing systemic immunity and mucosal immunity, and long-lasting immune response through a combined program of intramuscular vaccination of FMD vaccine and oral administration of D-galacto-D-mannan, an experiment was conducted according to the strategy described in Supplementary Figure 6A. The Exp group received vaccines with the same formula as the PC group *via* the same route, and administered orally 100 µg D-galacto-D-mannan/dose/mouse in 100 µL PBS. The vaccine formula for the Exp group and PC group was as follows: purified antigens type O (O PA2) and type A (A YC) (0.375 µg + 0.375 µg/dose), 10% $Al(OH)_3$, ISA 206 (Seppic; 50% w/w), 15 µg/dose/mouse Quil-A (InvivoGen), in a total volume of 100 µL. NC group received an equal volume of PBS. Mice were vaccinated *via* IM injection (0 dpv) and then orally administered D-galacto-D-mannan or PBS daily until 28 dpv. (**A**–**C**) experimental workflow (**A**); antibody titers, as determined using SP O ELISA (**B**); VN titers for O PA2, as determined using VN test (**C**). Data are presented as mean ± SEM of triplicate measurements (n = 5/group). Statistical analyses were performed using two-way analysis of variance followed by Tukey's *post-hoc* test. $^{**}p < 0.01$; $^{****}p < 0.0001$.
